# Endometriosis in a kidney with focal xanthogranulomatous pyelonephritis and a perinephric abscess

**DOI:** 10.1186/s13104-015-1574-1

**Published:** 2015-10-21

**Authors:** Chiu-Hsuan Cheng, Hann-Chorng Kuo, Borcherng Su

**Affiliations:** Department of Pathology, Buddhist Tzu Chi General Hospital, 707 Chung Yang Road, Section 3, Hualien, Taiwan; Department of Urology, Buddhist Tzu Chi General Hospital, 707 Chung Yang Road, Section 3, Hualien, Taiwan; School of Medicine, Tzu Chi University, Hualien, Taiwan

**Keywords:** Endometriosis, Xanthogranulomatous pyelonephritis, Kidney

## Abstract

**Background:**

The presence of endometriosis in the kidney is extremely rare. We report a case of endometriosis in renal parenchyma incidentally found in a malfunctioning kidney removed because of xanthogranulomatous pyelonephritis.

**Case presentation:**

A 53-year-old Chinese premenopausal woman presented with intermittent right flank pain for many years. Imaging studies revealed a contracted non-functioning right kidney and a perinephric abscess. The contracted kidney was considered to have resulted from chronic pyelonephritis. The abscess was drained. The patient subsequently underwent a right nephrectomy. Histology revealed endometriosis of renal parenchyma in addition to xanthogranulomatous pyelonephritis and a perinephric abscess. No evidence of endometriosis was identified at the pelvic site. The patient was symptom-free after operation.

**Conclusion:**

Endometriosis is a common benign condition in women of reproductive age that is characterized by the presence of endometrial glands and stroma outside the uterine cavity, which affects either genital or extragenital sites. Involvement of the urinary tract is rare. Among the urinary tract endometriosis, only a few cases involve the kidney. Renal endometriosis is difficult to diagnose; a final diagnosis relies on the pathohistologic findings. Treatment involves hormonal manipulation or a hysterectomy with bilateral salpingo-oophorectomy. Whether a nephrectomy required depends on the level of renal function. Although extremely rare, renal endometriosis should be part of the differential diagnostic spectrum when a contracted, non-functioning kidney is present. Early diagnosis might have prevented an unnecessary nephrectomy in cases of uncomplicated renal endometriosis.

## Background

Endometriosis is defined as the presence of normal endometrial mucosa abnormally implanted in locations other than the uterine cavity [1]. This common condition of reproductive-age females may affect pelvic or extrapelvic locations [1]. Renal endometriosis is an extremely rare condition with less than 25 cases reported previously to our knowledge. It presents with a space occupying lesion in the affected kidney [2–4] and is not a common etiology of atrophic kidney. Herein, we present a case of the renal parenchymal endometriosis in a contracted kidney removed for mal-functioning.

## Case presentation

A 53-year-old Chinese premenopausal woman, who had hypertension and diabetes mellitus under treatment, visited our hospital urology out-patient department (OPD) for intermittent recurrent right flank pain that had lasted for several years. She had not experienced hematuria. She denied any surgical history including gynecological operations. She had ever visited another clinic and was informed that she had bilateral renal stones. She had been treated for acute pyelonephritis.

At our urologic OPD, she appeared ill and had a mild fever. A physical examination revealed bilateral costovertebral angle tenderness. A urine analysis presented glycosuria (4+), pyuria [20 white blood cells (WBC) per high power field and WBC esterase of 2+, or 250 Leu/μL], and bacteriuria (1+). The blood urea nitrogen (BUN) was 16 mg/dL and the serum creatinine level was 1.2 mg/dL. An abdomen kidneys, ureters and bladder (KUB) X-ray showed bilateral renal stones. A bedside ultrasonogram showed a contracted right kidney in addition to bilateral stones. She was admitted under the impression of acute-on-chronic pyelonephritis of the right kidney.

**Fig. 1:**
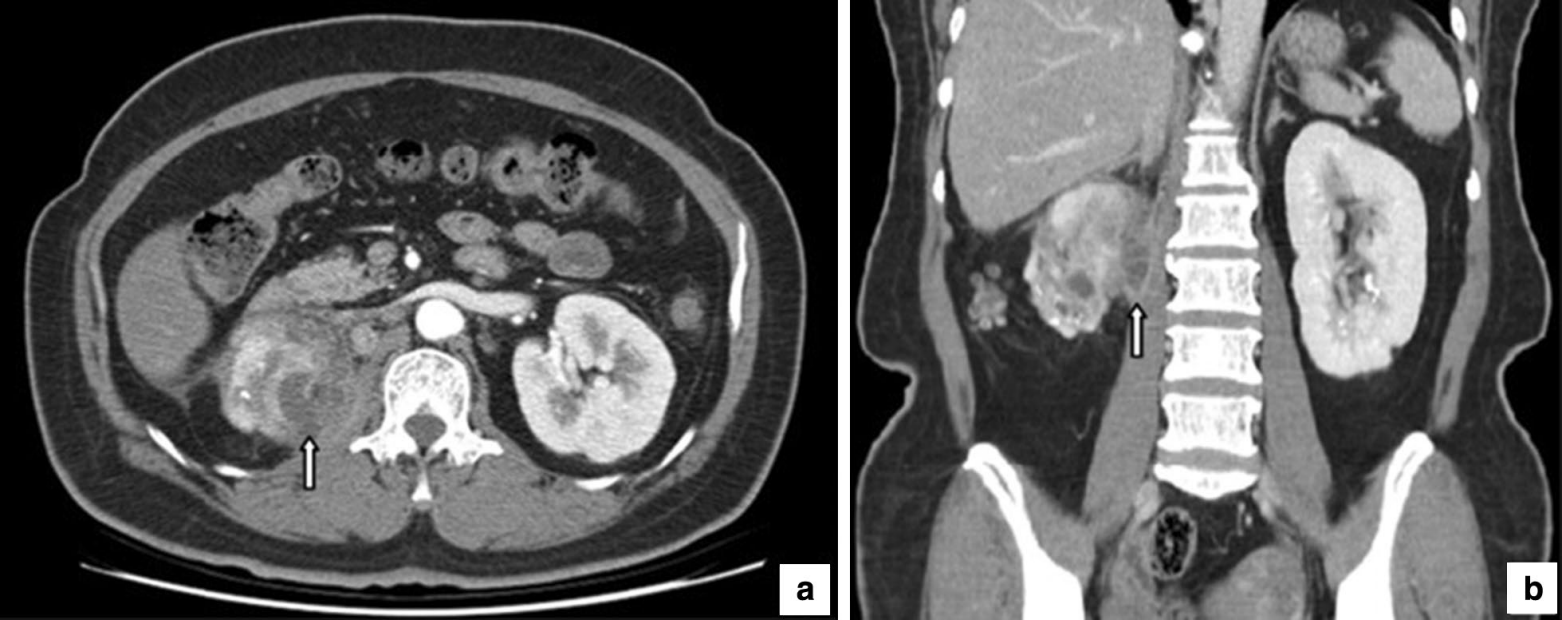
Contrast computerized tomography images. It shows the replacement of the right renal tissue by several rounded, low density areas. A perinephric abscess is noted invading the right psoas muscle (*arrows*). Renal stones, the frequent etiology of xanthogranulomatous pyelonephritis, are also seen. **a** Transverse section. **b** Coronal section

Systemic antibiotic therapy with cephradine was initiated. An abdomen to pelvic computerized tomography (CT) scan with contrast showed an abscess formation in the right kidney with invasion to the right psoas muscle. Compared with the left kidney, the right kidney was contracted and scarred. Several rounded, low-density areas surrounded by an enhanced rim of contrast and renal stones were noted (Fig. 1).

CT-guided pig-tail insertion was performed for drainage of the perinephric abscess. Both the blood and pus cultures grew *Citrobacter koseri.* The antibiotic was shifted to flomoxef sodium according to culture and sensitivity reports. The Tc-99m DTPA renal scintigraphy for glomerular filtration rate (GFR) estimation revealed that the GFRs of the right and left kidneys were 13.56 and 53.91 mL/min, respectively. The total scaled GFR was 71.03 mL/min. Therefore, she received a right nephrectomy 3 days after drainage. Intraoperative findings were an atrophic right kidney with a perirenal abscess and severe adhesion of the right kidney to the inferior vena cava and duodenum.

**Fig. 2:**
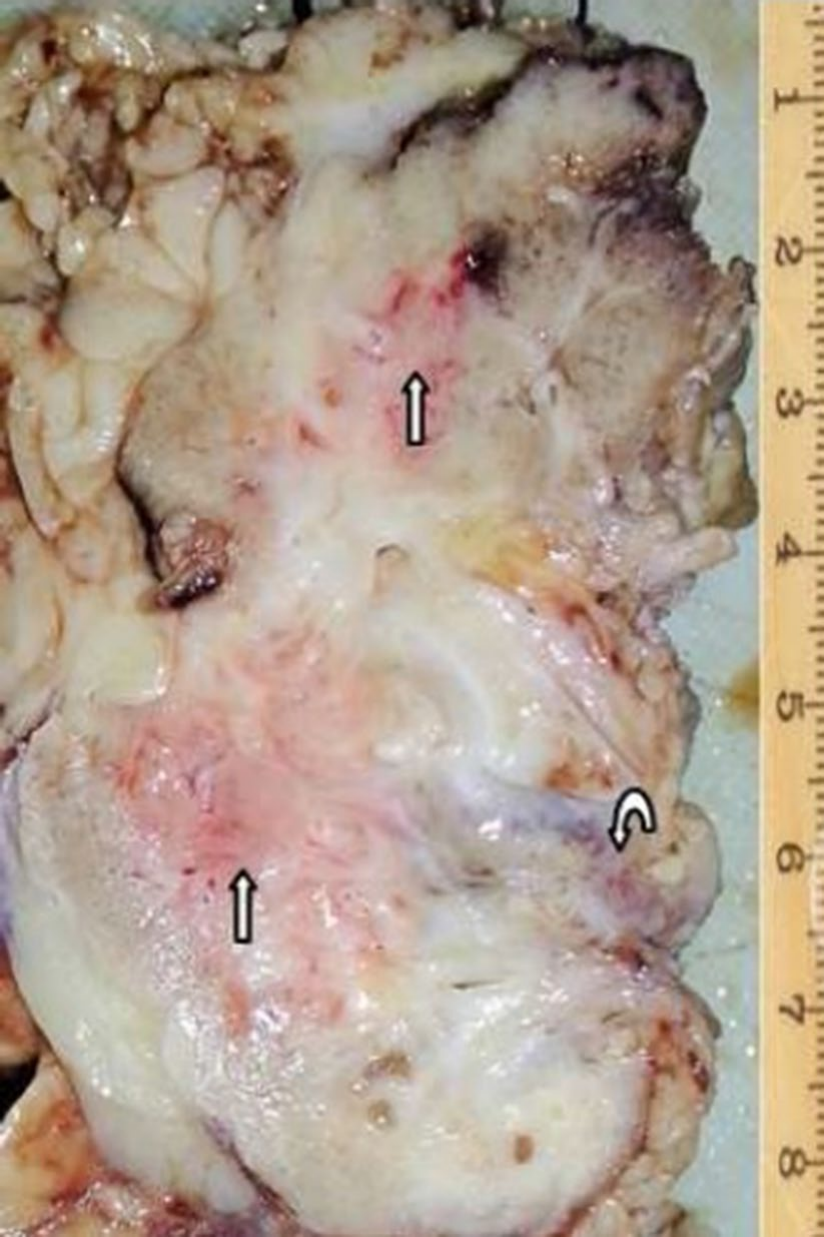
Gross appearance of the bisected kidney. The cortical surface is irregular and contracted. The renal parenchyma has multiple erythematous patches (*straight arrows*). The *curve arrow* marks the renal pelvic. These patches are compatible with hypodense areas seen in the contrast computed tomographic image and microscopically show endometriosis (The abscess, presents at deeper level, is not shown in this gross picture.)

**Fig. 3:**
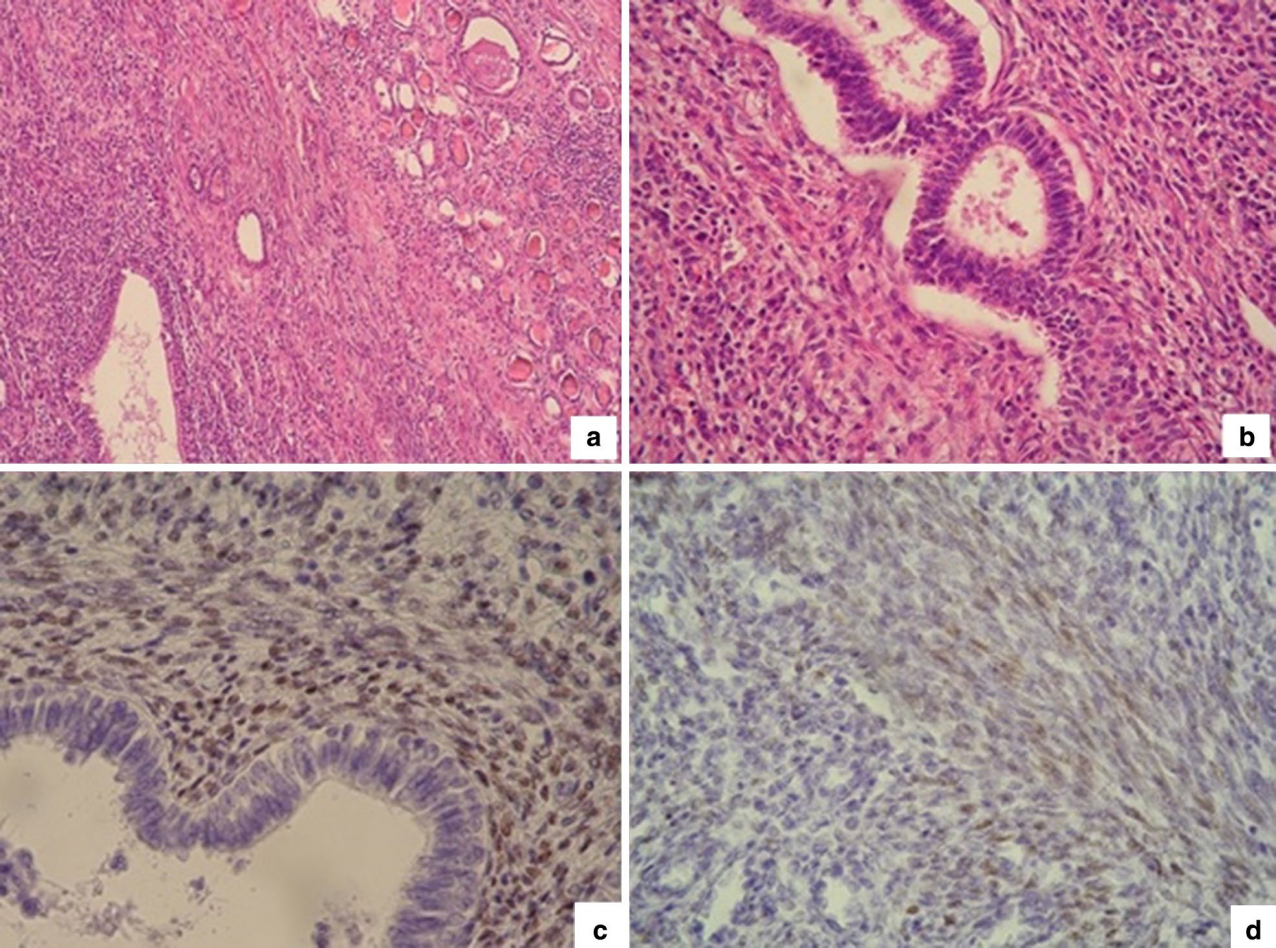
Photomicrographs of sections of kidney. **a** Endometrial glands and stroma with thyroidization of adjacent tubules and chronic inflammatory infiltrates in the interstitium (hematoxylin and eosin stain H&E, ×100 magnification), **b** endometrial glands, stromal cells and spiral arterioles (hematoxylin and eosin stain H&E, ×200 magnification), **c** positive estrogen receptor staining in stromal cell nuclei (immunohistochemical method IHC, ×200 magnification), **d** positive progesterone receptor staining in stromal cell nuclei (Immunohistochemical method IHC, ×200 magnification)

**Fig. 4:**
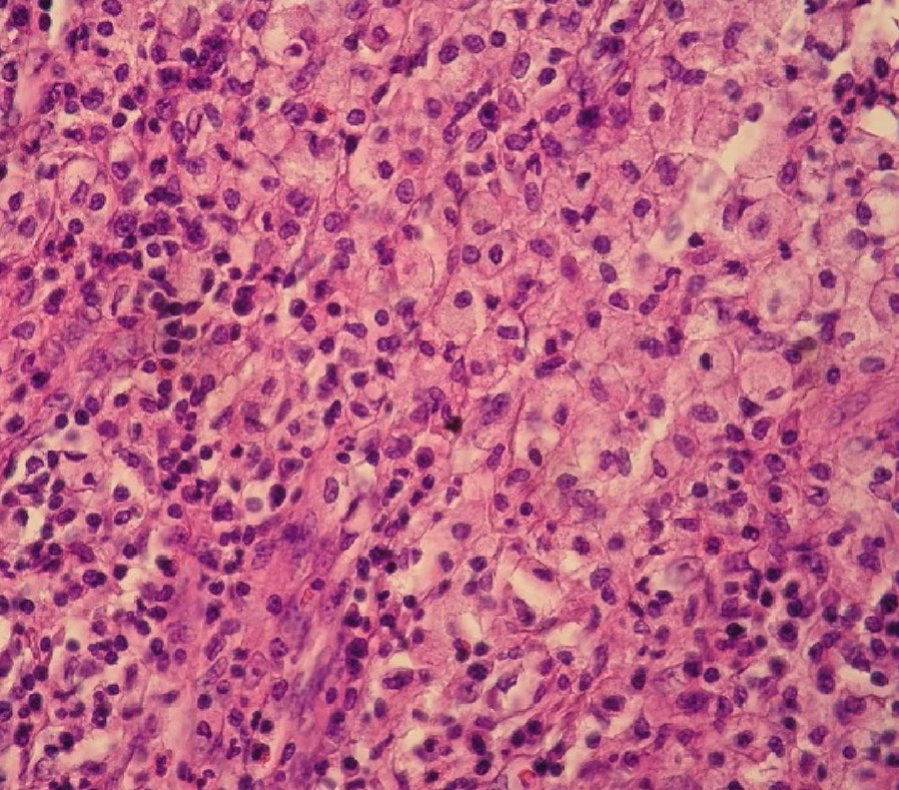
Xanthogranulomatous pyelonephritis (hematoxylin and eosin stain H&E ×200 magnification)

The resected right kidney was 8.0 cm long, 4.0 cm wide and 5.0 cm thick. The weight was 120 g. The bisected surface revealed irregular erythematous patchy lesions that blurred the corticomedullary junction (Fig. 2). There was also an abscess cavity with pus invading perirenal fat. Endometriosis of the right kidney was diagnosed histologically by the presence of endometrial glands and stromal tissue within the renal parenchyma with a few foci of microcalcification. Stromal cells and epithelial cells were focally positive for estrogen (ER) and progesterone receptors (PR) immunostains. Tubules within and adjacent to the endometrial tissue showed epithelial atrophy, luminal dilatation and thyroidization. Some of them were surrounded by lymphoid follicles (Fig. 3). Glomeruli were relatively spared. One area showed xanthogranulomatous pyelonephritis (XPN) characterized by granulocytes in the center surrounded by sheets of lipid-laden macrophages mixed with lymphocytes and plasma cells (Fig. 4). No endometrial elements were observed in the area of XPN.

Postoperatively, the patient recovered without any complications. She completed a 10-day course of intravenous flomoxef sodium. She was then discharged from the hospital with oral ceftibuten for 7 days. No other foci of endometriosis were identified apart from the right kidney by CT scan. The patient was symptom-free at the first week and first month follow-up visits.

## Discussion

Endometriosis is an important clinicopathological entity that is defined as the growth of endometrial tissue outside the endometrial cavity [1, 5]. It is the second most common pathologic condition in the female pelvis and is estimated to affect 15 % of women of reproductive age [3, 5]. Although the pelvis is the most commonly affected region, endometriosis can also occur at extrapelvic sites. In patients with extragenital lesions, the median age for diagnosis (35–40 years), is approximately 5 years older than that for diagnosis of genital lesions [2]. Extragenital endometriosis, despite its rarity, can be found at almost every site, including the intestines, urinary tract, abdominal scars, thorax, umbilicus, and kidneys, in that order [1, 2]. A small percentage of endometriotic cases (0.1–1 %) occur in the urinary tract, despite subclinical disease being potentially underestimated [5]. The locations and relative frequencies of urinary tract endometriosis are as follows: the bladder, 80–84 %; ureter, 14 %; kidney, 4 %; and urethra, 2 %; at an approximate ratio of 40:5:1:1, respectively [3, 6].

The pathogenesis of endometriosis is controversial, and the etiology is multifactorial. Despite numerous studies on endometriosis, the mechanism of occurrence at extragenital sites remains unclear. Three types of theories have been proposed to explain this: embryonic, migratory, and immunologic theories [3, 5, 7]. Embryonic theories suggest that endometriosis results from metaplastic changes of Wolffian, Mullerian, and occasionally peritoneal (celomic) structures [3, 5–7]. Migratory theories propose that retrograde menstruation, lymphovascular metastasis, and direct extension allow the endothelial cells to transplant into ectopic sites [2, 3, 5, 7]. Immunologic theories suggest that a suboptimal immune response may result in ectopic endometrial implantation [3, 5].

Blum and Frunhling advocated that endometrial structures develop through the metaplasia of renal tissue that is disturbed by the process of chronic inflammation [8]. In our patient, areas adjacent to endometrial structures showed chronic tubulointerstitial inflammation. However, endometrial tissue and XPN were found in separate areas. We assume that the inflammation was the result of endometriosis rather than the cause. A probable explanation for our case is the primary development of endometrial tissue via celomic metaplasia. Celomic membrane-related cells are disintegrated into the developing kidneys and subsequently stimulated by reproductive hormones [2].

Common manifestations of renal endometriosis are local pain and, rarely, cyclical hematuria, which is more common in ureteric and bladder endometriosis. Most patients have normal physical examination unless other pathologic conditions are also present. Renal endometriosis typically manifests insidiously and might be present for many years before the diagnosis was made. Sometimes the lesion may be completely asymptomatic and diagnosed only after nephrectomy for other presumed condition such as carcinoma [1, 2]. The present case underwent a nephrectomy because of renal malfunctioning.

Imaging techniques can be used to confirm a clinical diagnosis, but they are rarely conclusive for endometriosis. Transabdominal ultrasonography during menstruation may identify bladder endometriosis lesions [7]. CT and MRI are used to define the magnitude of the lesions and depth of invasion, but MRI demonstrates greater specificity in the detection of urinary tract endometriosis involving the bladder or ureter [9]. Regarding the kidney, CT images of previously reported renal endometriosis have indicated multiple focal hypo-dense areas of various sizes [2, 10]. The images of our patient yielded similar results. However, endometriosis may not be considered because such findings are also found in chronic pyelonephritis which is more common clinical condition than endometriosis.

The final diagnosis is generally made by a pathologist, who can identify endometrial glands and stroma in the specimen. Histological diagnosis is typically straightforward and can be made when only one of the endometrial glands or stroma is present [11]. Immunohistochemical staining is not generally required but may be helpful in diagnostically challenging cases. Regarding the immunoprofile of ureteral endometriosis, one study revealed a strong expression of epithelial cells for ER, PR, cytokeratin 7 (CK7), and cancer antigen 125 (CA125), in addition to cluster of differentiation 10 (CD10) expression within stroma [5]. Endometriosis at other sites would have a similar profile.

In previously recorded cases, the affected kidneys of patients with renal endometriosis were enlarged, either from a renal mass or from a space-occupying lesion [2–4, 8]. However, our patient presented with a contracted kidney, which probably resulted from either associated pyelonephritis or endometriosis per se. XPN is a variant of subacute to chronic pyelonephritis characterized by a severe pyelocalyceal inflammation, with prominent, foamy, lipid-laden macrophages. Grossly, the most characteristic changes are alterations of the calyces by yellow, friable material along with discrete abscesses. The histology of chronic pyelonephritis is non-specific, and it is one of many causes of such pattern of injury termed chronic tubulointerstitial nephritis [12].

We did not observe the classic gross picture of XPN in our patient. Instead, we found multiple erythematous patches, which were histologically proved to be endometriosis. However, the histologic slide that indicated XPN had no endometrial tissue. Therefore, it is appropriate to conclude that endometriosis and pyelonephritis were separate processes in our case. The endometriosis was more diffused, whereas XPN was a localized finding.

Endometriotic lesions often elicit a fibrotic reaction that can lead to adhesions around the focus and rarely foci of dystrophic calcification can be present in long standing endometriotic ovarian cysts [11]. Such lesions are also prone to subsequent bacterial infection. Regarding the kidneys, the association of endometriosis with chronic pyelonephritis including XPN, was described by Tore Gauperaa and Helge Stalsberg [8]. In our case, endometriosis might be an incidental finding, although it might also contribute to local tissue destruction and scarring.

There is little research on the management of renal endometriosis. The choice of treatment depends on the kidney condition, symptoms severity, extent of the disease, age of the patient, and whether pregnancy is planned [2, 3, 6]. Hormonal manipulation therapy can involve any of the following agents: danazol, GnRH agonist, medroxypregnisolone, estrogen-progestin combinations, and progestin alone. Hormonal therapy is optimal for patients of child-bearing age who wish to retain reproductive capability and for those with normal renal function [2]. If the patient does not want to become pregnant, a total abdominal hysterectomy with bilateral salpingo-oophorectomy can be performed with or without adjuvant hormonal therapy. Whether a nephrectomy is required depends on the level of renal function [2].

Although endometriosis is a benign condition, a high index of suspicion is necessary for diagnosis, especially at unusual sites such as the kidney, because of the high local aggressiveness of tissue destruction [13]. Early diagnosis might have prevented an unnecessary nephrectomy in cases of uncomplicated renal endometriosis. Endometriosis also bears a high risk of recurrence [13]. Therefore, a close follow-up is required.

## Conclusion

In conclusion, renal endometriosis should be part of the differential diagnostic spectrum when a contracted, non-functioning kidney is present. Diagnosis generally relies on pathohistologic findings.

## Consent

Written informed consent was obtained from the patient for publication of this Case report and any accompanying images.

## Abbreviations

BUN: blood urea nitrogen
CA 125: cancer antigen 125
CD 10: clusters of differentiation 10
CK 7: cytokeratin 7
cm: centimeters
CT: computed tomography
ER: estrogen receptor
g: grams
GFR: glomerular filtration rate
H&E: hematoxylin and eosin stain
IHC: immunohistochemical method
KUB: kidneys, ureters and bladder x-ray
MRI: magnetic resonance imaging
OPD: out-patient department
PR: progesterone receptor
Tc99m-DTPA: Tc99m-diethylene triamine pentacaetic acid
WBC: white blood cells
XPN: xanthogranulomatous pyelonephritis
